# Prevalence of Ménière's Disease in Syrian Patients with hypothyroidism: Cross-sectional study

**DOI:** 10.1016/j.amsu.2022.104405

**Published:** 2022-09-02

**Authors:** Anan Bakdounes, Nawal Akashe, Mhd Obai Alchallah, Homam Alolabi, Duaa Bakdounes, Fatema Mohsen, Louei Darjazini Nahas

**Affiliations:** a Faculty of Medicine, Syrian Private University, Damascus, Syria; b Department of Surgery Division of Otorhinolaryngology, Faculty of Medicine, Syrian Private University, Damascus, Syria

**Keywords:** Autoimmunity, Endolymph, Endocrine disorder, Labyrinth, War, MD, Ménière's Disease, PTA, Pure Tone Audiometry, TSH, Thyroid-Stimulating Hormone, ENT, Ear Nose Throat, SD, standard deviations, IRB, Institutional Review Board, DM, Diabetes Mellites, TNFα, Tumor Necrosis Factor α, IL1, Interleukin 1, IL6, Interleukin 6

## Abstract

**Background:**

Ménière's Disease, a long-term debilitating disorder has been increasingly found among patients with hypothyroidism. Our study aims to evaluate the prevalence of ménière's disease among hypothyroid patients and assess the interrelationship between patients' symptomology and ménière's disease.

**Materials and methods:**

A cross-sectional study was performed at the endocrinology clinics at Damascus Hospital and Syrian Red Crescent Hospital, Damascus, Syria between September 2021 and January 2022. Patients with hypothyroidism were interviewed using a questionnaire. The questionnaire contained questions about socio-demographic information, hypothyroid history, diagnostic criteria of ménière's disease, chief complaint, medical history, and lab test results. Patients, who reported ménière's disease symptoms, were referred to the otorhinolaryngology clinic for confirmation or exclusion of ménière's disease. At the clinic, patients underwent an otoscopy and a pure tone audiometry, probable and definite ménière's disease was diagnosed accordingly.

**Results:**

Of 217 hypothyroid patients included in the sample, 17 (7.8%) were diagnosed with definite ménière's disease and 31 (14.3%) were diagnosed with probable ménière's disease. Hypothyroid symptoms reported among patients diagnosed with definite ménière's disease compared to no diagnosis differed by feeling low (χ2 (1, 217) = 4.014, p = 0.045), and depressive appearance (χ2 (1, 217) = 8.887, p = 0.003). Patients diagnosed with definite ménière's disease, probable ménière's disease, and both definite and probable ménière's disease were more likely to report that their symptoms affected their lifestyle compared to those that reported no effect (χ2 (3, 217) = 62.565, p < 0.001), (χ2 (3, 217) = 31.380, p < 0.001), and (χ2 (3, 217) = 35.542, p < 0.001), respectively.

**Conclusion:**

A high number of hypothyroid patients were diagnosed with MD. Clinicians should consider clinically screening for MD among hypothyroid patients presenting to clinics.

## Highlights

• This study shows a significant association between hypothyroidism and Ménière's disease.
• Rates of feeling low and depressive appearance increase in definite MD patients.
• Meniere's disease patients were predisposed to the disease's effect on their lifestyle.

## 1 Introduction

Ménière's disease (MD), a debilitating disorder that affects the membranous labyrinth of the inner ear, was described by Prosper Ménière in 1861 and is diagnosed clinically by recurrent episodes of vertigo along with cochlear symptoms of low or medium frequency sensorineural hearing loss, tinnitus, and/or ear fullness [1]. A previous study revealed that the average annual prevalence of MD was 34.5% and the average annual incidence of MD was 5.0 per 100,000 populations [2]. The overall incidence of MD was found to be significantly higher in a hypothyroidism cohort 8.65 per 1000 person-years versus a non-hypothyroidism cohort 6.38 per 1000 person-years [3].

MD is classified into two categories: definite MD, and probable MD. The diagnosis of definite MD is based on episodic vertigo associated with low to medium frequency sensorineural hearing loss recorded on pure tone audiometry (PTA) and fluctuating auditory symptoms (tinnitus, and/or fullness) in the affected ear [4]. The duration of vertigo spells ranges from 20 min to 12 h. Probable MD is identified by occasional vestibular symptoms (vertigo or dizziness) associated with fluctuating aural symptoms lasting between 20 min and 24 h [4]. The aetiology of MD remains unknown; however multiple factors have been blamed including immunologic disease, psychological factors, infections, trauma, genetic predisposition, metabolic disorders, and hormone dysfunction [5].

Thyroid dysfunction is the most common endocrine disorder [6]. Hypothyroidism is the failure of the thyroid gland to adequately secrete thyroid hormones [7]. The clinical presentation of thyroid disease is variable and nonspecific and constitutes a wide spectrum of clinical features [8]. Thyroid failure results in primary hypothyroidism [9]. Hypothyroidism is due to a deficiency in thyroid hormones. Predisposition and pathophysiology are thought to influence the association between autoimmune thyroid disease and many other autoimmune diseases [10,11]. While the prevalence of autoimmune thyroid diseases, is estimated to occur in approximately 1–5% of the general population, Hashimoto's thyroiditis is the most common cause of hypothyroidism and account for 47% [12]. This supports the theory that hypothyroidism contributes to autoimmune disease. Moreover, the abnormal metabolisms of patients with thyroid disease could stimulate the endolymphatic hydrops and MD. [] Hypothyroidism probably changes the composition of endolymphatic fluid through the spread of thyroid autoantibody complexes in the endolymph [13,14]. The measurement of thyroid-stimulating hormone (TSH) is widely used to diagnose hypothyroidism, once diagnosed with hypothyroidism, patients require mandatory lifetime therapy with oral levothyroxine [9].

The Syrian war and the resulting humanitarian crisis have drastically affected the healthcare of civilians. The demand for medicines and medical support has impacted the quality of healthcare Syrians receive. More than 90% of medicines were locally manufactured, before the conflict in Syria. Sadly, the effects of economic sanctions, destruction of pharmaceutical plants and storage facilities for imported medicines, currency crisis, and an increase in operational costs have negatively impacted the production of medicines. As a result, local production of medicines has been reduced to 10%. Therefore, the World Health Organization has evaluated the need for essential medicines, including the need for thyroid hormones, and levothyroxine [15,16]. Currently, there are four thyroxine supplement brands available, the imported brand includes Euthyrox, and nationally manufactured brands include Levothyroxine, Syntroxine asia, and Eltroxin available in dosages: 100 μg, 50 μg, and 25 μg, 100 tablets. Additionally, patients are required to self-fund their lifetime treatment and consultations.

Since both hypothyroidism and MD share a common pathophysiology of autoimmunity, former studies have proved the relationship between hypothyroidism and MD [3,13,17]. Other studies found hearing impairment in patient with Pendred syndrome as well as patients with acquired hypothyroidism [18]. People with hypothyroidism had a hearing loss rate of 43%. Tinnitus was found in 7% of cases and vertigo in 29.1% of cases. The incidence of these symptoms was linearly correlated with the severity of hypothyroidism [19]. Further reports have proved an improvement in patients' MD symptoms when treated appropriately with thyroxine [7,19]. However, none have screened for MD among hypothyroid patients. The aims of this study included: (1) evaluate the prevalence of MD among hypothyroid patients; (2) assess the interrelationship between patients' symptomology and MD; (3) determine the severity of MD on hypothyroid patient's lifestyle.

## 2 Materials and Methods

### 2.1 Study design, setting, and participants

A cross-sectional study was performed at the endocrinology clinics at Damascus Hospital and Syrian Red Crescent Hospital, Damascus, Syria between September 2021 and January 2022. All patients diagnosed with hypothyroidism, who agreed to participate, were included in the study. Criteria of exclusion were a history of cerebrovascular accident, panhypopituitarism, brain tumours, or otologic diseases such as otitis media and tympanic membrane perforation. Written informed consent was obtained from patients over the age of 18 years, while informed consent was sought for patients under 18 years of age from the patient and guardian, and the interview was conducted in the presence of the patient's guardian.

### 2.2 Data collection and procedures

Of 217 patients, who were diagnosed by an endocrinologist with hypothyroidism, attending their regular endocrinology clinic appointment, were interviewed using a questionnaire created by the authors. The questionnaire contained questions about socio-demographic information (such as gender, age, accommodation, work and education status, and smoking), hypothyroid history, diagnostic criteria of MD, chief complaint, medical history, and lab test results. Additionally, questions were included to exclude all differential diagnoses of MD [4].

#### 2.2.1 Diagnosis of ménière disease

The diagnostic criteria of MD include recurrent episodes of vertigo lasting from 20 min to 12 h, tinnitus, low- or mid-frequency sensorineural hearing loss, and ear fullness [4]. Patients, who reported MD symptoms, were referred for a same-day appointment at the otorhinolaryngology clinic. At the clinic, patients underwent an otoscopy to rule out contraindications such as tympanic membrane perforation or ear wax for a pure PTA. Treatment was prescribed for patients who had ear wax and were scheduled for a follow-up appointment with the clinic to monitor treatment and then referred for PTA. Of 48 patients referred to the otorhinolaryngology clinic, 23 patients did not consent to PTA. Depending on the PTA results patients were assessed by an Ear Nose Throat (ENT) specialist and divided into probable MD and definite MD. Definite MD was diagnosed based on the presence of low to mid-frequency sensorineural hearing loss. Probable MD was diagnosed based on symptoms, with or without a normal PTA result.

The work in this study complies with the principles laid down in the Declaration of.

Mathew G and Agha R, for the STROCSS Group. STROCSS 2021: Strengthening the Reporting of cohort, cross-sectional and case-control studies in Surgery. International Journal of Surgery 2021; 96:106,165 [20].

### 2.3 Statistical analysis

Data were displayed as frequencies and percentages for categorical variables, and means with standard deviations (SD) for continuous variables. The Statistical Package for Social Sciences version 25.0 (SPSS Inc., Chicago, IL, United States) was used to analyze the study. The chi-square test was used to compare hypothyroid symptoms against probable MD, definite MD, and both probable and definite MD. Additionally, the chi-square test was performed to examine the relation between MD and its effect on patients' lifestyles. Students' independent *t*-test was used to study the relation between MD and TSH levels. Statistical significance was considered at a p-value <0.05.

### Ethical statement

Ethical approval was obtained from the Institutional Review Boards (IRB) of the Faculty of Medicine at the Syrian Private University, Damascus Hospital, and Syrian Red Crescent Hospital. No reference number was given.

### 2.4 Registration of research studies

1. Name of the registry: Prevalence of Ménière's Disease in Syrian Patients with Hypothyroidism.
2. Unique Identifying number or registration ID: 8157.
3. Hyperlink to your specific registration (must be publicly accessible and will be checked):
  https://www.researchregistry.com/browse-the-registry#home/

## 3 Results

### 3.1 Socio-demographic characteristics of patients

Of 217 patients included in the sample, 204 (94%) were females, and 13 (6%) were males, with a mean age of 40.4 (±14.6) years. The ages ranged from 8 to 79 years, and the median age was (40) years. The mean BMI was 27.6 kg/m^2^, and 61 (28.1%) smoke. Unemployed patients represented the majority 128 (59.0%), while non-educated represented the minority 25 (11.0%), respectively (Table 1).

**Table 1:** Demographic characteristics of patients.

Table 1. Demographic characteristics N= 217		
**Group**	**Categories**	**N (%)**
**Gender**	**Male**	13 (6)
	**Female**	204 (94)
**Age**	**8–13**	12 (5.5)
	**14–17**	6 (2.8)
	**18–21**	4 (1.8)
	**22− 42**	98 (45.2)
	**43–50**	42 (19.4)
	**51–79**	55 (25.3)
**Accommodation**	**City**	120 (55.3)
	**Suburb**	97 (44.7)
**Work status**	**Don't work**	128 (59.0)
	**Student**	22 (10.1)
	**Full time job**	40 (19.4)
	**Part time job**	18 (8.3)
	**Retired**	7 (3.2)
**Education**	**Non- educated**	25 (11.5)
	**Primary**	42 (19.4)
	**Elementary**	55 (25.3)
	**Senior high**	30 (13.8)
	**University/institute**	62 (28.1)
	**Postgraduate**	4 (1.8)
**Smoking**	**Cigarette and Water pipe**	5 (2.3)
	**Cigarette only**	32 (14.7)
	**Water pipe only**	24 (11.1)
	**Previous smoker**	6 (2.8)
	**Non smoker**	150 (69.1)

### 3.2 Clinical characteristics of hypothyroid patients

Common hypothyroid symptoms include tiredness 156 (71.9%), pale skin 144 (66.4%), respiratory distress 141 (65%), lateral hair loss 137 (63.1%), numbness 136 (62.7%), cold intolerance 133 (61.3%), feeling low 129 (59.4%), dry skin 129 (59.4%), dementia 121 (55.8%), headache 118 (54.4%), jaundice skin 117 (53.9%), arrhythmias 115 (53.0%), and drowsiness 111 (51.2%). Shockingly, 51 (23.5%) reported non-compliance with levothyroxine therapy, the reasons behind the non-compliance, include neglection, costs, forgetfulness, and unhappiness with the medications directions of use (Table 2)

**Table 2:** Clinical characteristics of hypothyroid patients.

Table 2. Clinical characteristics of hypothyroid patients N= 217	
**Symptoms**	N (%)
**Commitment to the medicine**	166 (76.5)
**Weight gain**	100 (46.1)
**Loss of appetite**	57 (26.3)
**Cold intolerance**	133 (61.3)
**Lack of sweating**	79 (36.4)
**Drowsiness**	111 (51.2)
**Respiratory distress**	141 (65.0)
**Chest pain**	85 (39.2)
**Tiredness**	156 (71.9)
**Headache**	118 (54.4)
**Feeling low**	129 (59.4)
**Lateral hair loss**	137 (63.1)
**Constipation**	77 (35.5)
**Menstrual disturbance**	70 (32.3)
**Arrhythmias**	115 (53.0)
**Numbness**	136 (62.7)
**Memory loss**	121 (55.8)
**Tongue enlargement**	56 (25.8)
**Dry skin**	129 (59.4)
**Rough and split hair**	101 (46.5)
**Pale skin**	144 (66.4)
**Vitiligo**	6 (3.8)
**jaundiced skin**	117 (53.9)
**Unkept appearance**	70 (32.3)

### 3.3 Prevalence of MD symptoms

**Fig. 1:**
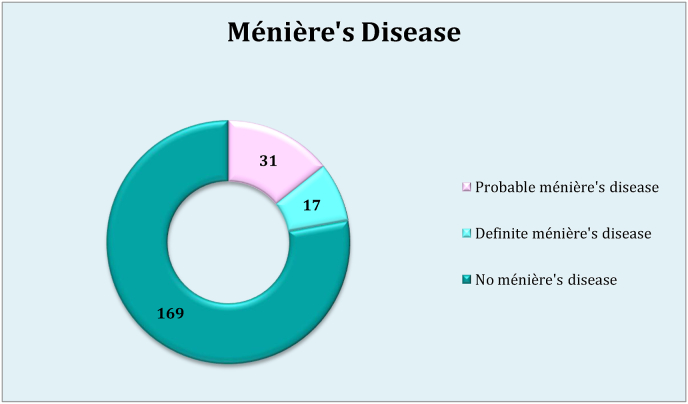
Distribution of ménière's disease among patients.

MD symptoms include vertigo 117 (53.9%), tinnitus 87 (40.1%), ear fullness 56 (25.8%), and hearing loss 53 (24.4%) (Table 3). The prevalence of MD was 48 (22.1%), probable MD and definite MD was 31 (14.3%) and 17 (7.8%) respectively (Fig. 1).

**Table 3:** Prevalence of MD symptoms among hypothyroid patients.

Table 3. Table 3. Prevalence of MD symptoms among hypothyroid patients N= 217		
**Symptoms**	group	N (%)
**Vertigo**	**Yes**	117 (53.9)
	**No**	100 (46.1)
**Duration of dizziness**	**Seconds**	56 (25.8)
	**Minutes**	47 (21.7)
	**One hour**	3 (1.4)
	**Couple of hours**	2 (0.9)
	**Day**	2 (0.9)
	**Couple of days**	7 (3.2)
**Time between vertigo events**	**Hour**	6 (2.8)
	**One day**	21 (9.7)
	**Days**	5 (2.3)
	**Week or more**	81 (37.3)
**Balance**	**Loss of balance**	92 (42.4)
	**Fall down**	7 (3.2)
**Tinnitus**	**Yes**	87 (40.1)
	**No**	130 (59.9)
**Frequency of tinnitus**	**High**	30 (13.8)
	**Low**	57 (26.3)
**Effect of tinnitus on patient**	**Low**	65 (30.0)
	**Moderate**	16 (7.4)
	**Severe**	6 (2.8)
**Effect of tinnitus on quality of sleep**	**wake from sleep**	10 (4.6)
	**Don't wake from sleep**	76 (35.0)
**Hearing loss**	**Yes**	53 (24.4)
	**No**	164 (75.6)
**Evolution**	**Sudden**	14 (6.5)
	**Gradual**	35 (16.1)
	**Unsteady**	4 (1.8)
**Hearing loss onset**	**During dizziness**	23 (10.6)
	**Out of dizziness**	1 (0.5)
	**Not related with dizziness**	32 (14.7)
**Hearing loss duration**	**Seconds**	32 (14.7)
	**Minutes**	16 (7.4)
	**Hour**	2 (0.9)
	**More**	6 (2.8)
**Ear fullness**	**Yes**	56 (25.8)
	**No**	161 (74.2)
**Eye symptoms**	**Yes**	112 (51.6)
	**No**	105 (48.4)
**Headache**	**Yes**	111 (51.2)
	**No**	106 (48.8)
**How does these symptoms affect your life**	**No effect**	143 (65.9)
	**Low**	34 (15.7)
	**Moderate**	29 (13.4)
	**Severe**	11 (5.1)

### 3.4 Association between symptoms and MD

#### 3.4.1 Association between hypothyroid symptoms and probable MD

Hypothyroid symptoms reported among patients diagnosed with probable MD compared to no diagnosis were significantly associated with the following: weight gain (χ2 (1, 217) = 6.828, p = 0.009), cold intolerance (χ2 (1, 217) = 3.966, p = 0.046), respiratory distress (χ2 (1, 217) = 5.673, p = 0.017), chest pain (χ2 (1, 217) = 18.225, p < 0.001), headache (χ2 (1, 217) = 12.681, p < 0.001), arrhythmia (χ2 (1, 217) = 6.524, p = 0.011), numbness (χ2 (1, 217) = 9.222, p = 0.002), and memory loss (χ2 (1, 217) = 4.982, p = 0.026) (Table 4).

**Table 4:** Association between patients' hypothyroidism symptoms and ménière's disease.

Table 4. Association between patients’ hypothyroidism symptoms and ménière’s disease												
Symptoms	Probable ménière's disease		*X*^*2*^	*p-* value	Definite ménière's disease		*X*^*2*^	*p-* value	Ménière's disease		*X*^*2*^	*p-* value
	Yes N (%)	No N (%)			Yes N (%)	No N (%)			Yes N (%)	No N (%)		
**Commitment to the medicine**	23 (74.2)	143 (76.9)	0.107	0.744	6 (35.3)	160 (80)	17.418	**<.001**	29 (60.4)	137 (81.1)	8.865	**0.003**
**Weight gain**	21 (67.7)	79 (42.5)	6.828	**0.009**	9 (52.9)	91 (45.5)	0.349	0.555	30 (62.5)	70 (41.4)	6.686	**0.010**
**Loss of appetite**	9 (29.0)	48 (25.8)	0.143	0.706	6 (35.3)	51 (25.5)	0.776	0.378	15 (31.3)	42 (24.9)	0.790	0.374
**Cold intolerance**	24 (77.4)	109 (58.6)	3.966	**0.046**	12 (70.6)	121 (60.5)	0.672	0.412	36 (75.0)	97 (57.4)	4.883	**0.027**
**Lack of sweating**	11 (35.5)	68 (36.6)	0.013	0.908	5 (29.4)	74 (37.0)	0.390	0.532	16 (33.3)	63 (37.3)	0.251	0.616
**Drowsiness**	18 (58.1)	93 (50.0)	0.164	0.685	11 (64.7)	100 (50.0)	1.356	0.244	29 (60.4)	82 (48.5)	2.117	0.146
**Respiratory distress**	26(83.9)	115 (61.8)	5.673	**0.017**	14 (82.4)	127 (63.5)	2.447	0.118	40 (83.3)	101 (59.8)	9.126	**0.003**
**Chest pain**	22 (71.0)	63 (33.9)	18.225	**<.001**	8 (47.1)	77 (38.5)	0.482	0.488	30 (62.5)	55 (32.5)	14.078	**<.001**
**Tiredness**	26 (83.9)	130 (69.9)	2.569	0.109	15 (88.2)	141 (70.5)	2.439	0.118	41 (85.4)	115 (68)	5.581	**0.018**
**Headache**	26 (83.9)	92 (49.5)	12.681	**<.001**	12 (70.6)	106 (53)	1.954	0.162	38 (79.2)	80 (47.3)	15.266	**<.001**
**Feeling low**	19 (61.3)	110 (59.1)	0.051	0.821	14 (82.4)	115 (57.5)	4.014	**0.045**	33 (68.8)	96 (56.8)	2.213	0.137
**Lateral hair loss**	21(67.7)	116 (62.4)	0.330	0.566	11 (64.7)	126 (63.0)	0.020	0.889	32 (66.7)	105 (62.1)	0.331	0.565
**Constipation**	11 (35.5)	66 (35.5)	0.000	1	9 (52.9)	68 (34.0)	2.455	0.117	20 (41.7)	57 (33.7)	1.029	0.310
**Menstrual disturbance**	10 (32.3)	60 (32.3)	0.000	1	7 (41.2)	63 (31.5)	0.671	0.413	17 (35.4)	53 (31.4)	0.281	0.596
**Arrhythmias**	23 (74.2)	92 (49.5)	6.524	**0.011**	12 (70.6)	103 (51.5)	2.292	0.130	35 (72.9)	80 (47.3)	9.819	**0.002**
**Numbness**	27(87.1)	109 (58.6)	9.222	**0.002**	14 (82.4)	122 (61.0)	3.054	0.081	41 (85.4)	95 (56.2)	13.628	**<.001**
**Memory loss**	23 (74.2)	98 (52.7)	4.982	**0.026**	13 (76.5)	108 (54.0)	3.207	0.073	36 (75.0)	85 (50.3)	9.248	**0.002**
**Tongue enlargement**	8 (25.8)	48 (25.8)	0.000	1	4 (23.5)	52 (26.0)	0.050	0.823	12 (25.0)	44 (26.0)	0.021	0.885
**Dry skin**	22 (71.0)	107 (57.5)	2.485	0.115	11 (64.7)	118 (59.0)	0.212	0.646	33 (68.8)	96 (56.8)	2.213	0.137
**Rough and split hair**	14 (45.2)	87 (46.8)	0.028	0.868	11 (64.7)	90 (45.0)	2.445	0.118	25 (52.1)	76 (45.0)	0.760	0.383
**Pale skin**	20 (64.5)	124 (66.7)	0.055	0.815	14 (82.4)	130 (65.0)	2.113	0.146	34 (70.8)	110 (65.1)	0.553	0.457
**Vitiligo**	0 (0.0)	6 (3.2)	1.187	0.276	1 (5.9)	5 (2.5)	0.667	0.414	1 (2.1)	5 (3.0)	0.107	0.744
**jaundiced skin**	17 (54.8)	100 (53.8)	0.12	0.911	8 (47.1)	109 (54.5)	0.349	0.555	25 (52.1)	92 (54.4)	0.083	0.773
**Unkept appearance**	7 (22.6)	63 (33.9)	1.550	0.213	11 (64.7)	59 (29.5)	8.887	**0.003**	18 (37.5)	52 (30.8)	0.775	0.379

#### 3.4.2 Association between hypothyroid symptoms and definite MD

Hypothyroid symptoms reported among patients diagnosed with definite MD compared to no diagnosis were significantly differed by feeling low (χ2 (1, 217) = 4.014, p = 0.045), and depressive appearance (χ2 (1, 217) = 8.887, p = 0.003) (Table 4).

#### 3.4.3 Association between hypothyroid symptoms and the total MD

Hypothyroid symptoms reported among patients diagnosed with both probable and definite MD compared to no were significantly differed by weight gain (χ2 (1, 217) = 6.686, p = 0.010), cold intolerance (χ2 (1, 217) = 4.883, p = 0.027), respiratory distress (χ2 (1, 217) = 9.126, p = .003), chest pain (χ2 (1, 217) = 14.078, p < 0.001), tiredness (χ2 (1, 217) = 5.581, p = 0.018), headache (χ2 (1, 217) = 15.266, p < 0.001), arrythmia (χ2 (1, 217) = 9.819, p = .002), numbness (χ2 (1, 217) = 13.628, p < 0.001), and memory loss (χ2 (1, 217) = 9.248, p = .002) (Table 4).

### 3.5 Effect of MD on patients' lifestyle

Patients diagnosed with definite MD, probable MD, and both definite and probable MD were more likely to report that their symptoms affected their lifestyle compared to those that reported no effect (χ2 (3, 217) = 62.565, p < 0.001), (χ2 (3, 217) = 31.380, p < 0.001), and (χ2 (3, 217) = 35.542, p < 0.001), respectively (Table 5).

**Table 5:** Effect of ménière's disease on patient's lifestyle and TSH levels.

Table 5. Effect of ménière's disease on patient's lifestyle							
		No effect (%)	Mild (%)	Moderate (%)	Severe (%)	*X*^*2*^	*p-* value
**Ménière's disease**	**Yes**	11 (7.7)	11 (32.4)	18 (62.1)	8 (72.7)	62.565	**< .001**
	**No**	132 (92.3)	23 (67.6)	11 (37.9)	3 (27.3)		
**Probable ménière's disease**	**Yes**	9 (6.3)	6 (17.6)	13 (44.8)	3 (27.3)	31.380	**< .001**
	**No**	134 (93.7)	28 (82.4)	16 (55.2)	8 (72.7)		
**Definite ménière's disease**	**Yes**	2 (1.4)	5 (14.7)	5 (17.2)	5 (45.5)	35.542	**< .001**
	**No**	141 (98.6)	29 (85.3)	24 (82.8)	6 (54.5)		
**Effect of ménière's disease on TSH levels**							
		**TSH** **Mean (**± **SD)**	**t**	***p-* value**			
**Meniere disease**	**Yes**	10.2 (19.6)	0.420	0.675			
	**No**	8.9 (18.9)					
**Probable ménière's disease**	**Yes**	11.6 (24.1)	0.757	0.450			
	**No**	8.8 (18.1)					
**Definite ménière's disease**	**Yes**	7.7 (5.9)	0.336	0.737			
	**No**	9.3 (19.8)					

## 4 Discussion

The literature has repeatedly proven the association between hypothyroidism and MD [3,7,17,[21], [22], [23]]. A retrospective study containing 211 patients with classic MD where 208 patients were tested for hypothyroidism, revealed only one patient with an abnormal test result. They concluded that routine screening of thyroid function was not recommended among MD patients unless a history suggestive of metabolic disorder is present [24].

To the best of our knowledge, this first study tests for MD among patients with hypothyroidism presenting with aural symptoms. After testing for MD among hypothyroid patients presenting with aural symptoms, the prevalence of MD was found to be high (22.1%), probable MD was 14.3% and definite MD was 7.8%. Our findings are higher compared with a study conducted in Taiwan (5%) [3]. We believe that the high prevalence may be due to the lack of awareness among both patients and doctors about the disease and its association with hypothyroidism as well as the inadequate adherence to the medication from the patients.

MD can be associated with numerous comorbidities including Diabetes Mellitus (DM), hyperlipidaemia, hypertension, and cirrhosis [3,25,26]. However, the inflammatory or metabolic changes in patients with hypothyroidism may affect the inner ear inflammation and homeostasis of endolymphatic flow [17]. In addition, inflammatory cytokines such as tumor necrosis factor α (IFα) and interleukin 1 and 6 (IL1, IL6) decrease the expressions of sodium/iodine symporter, impeding the iodide uptake in the thyroid gland [27,28]. This inflammatory or degenerative alteration in the inner ear epithelia can also raise the likelihood of MD [17]. Therefore, the disturbance of this composition as a result of the altered metabolism could affect the vestibular functioning.

Definite MD patients were individually related to feeling low (82.4%) and depressive appearance (64.7%). A systematic review found that 50% of MD patients have varying degrees of depression [29]. Therefore, Regular assessment of depressive symptoms among MD patients facilitates early detection of critical cases. This permits a prompt diagnosis and therapy of depression to guarantees a lifelong quality of life for MD patients.

MD is a long-term disabling disease that not only impacts one's psychological wellbeing and physical functioning but also restricts the quality of life through stigmatization [30]. In our study, MD diagnosis was found to affect patients' lifestyles more compared with those who had no diagnosis. Former studies have used scales to assess the quality of well-being among patients with MD; results have shown severely incapacitated patients. Acute episodes of MD are the most debilitating condition endured by people who survive any illness [31]. Currently, there is no cure for MD; however, lifestyle changes can help prevent or reduce attacks [32].

In this study, many patients refused to undergo PTA to confirm their MD diagnosis and were unable to assess vestibular function tests. Thus, the MD endotypes involving degenerated distal endolymphatic sac and hypoplastic endolymphatic sac were indistinguishable. Although associations between MD type and severity may vary, evaluation of clinical otovestibular symptoms in MD is more predictable. Our findings illustrate an association between hypothyroid symptoms and both probable and definite MD. Herein screening for MD in hypothyroid patients is highly recommended, especially if thyroid hormone medication is not already taken. Plan to modify the diagnostic system and follow-up of patients with hypothyroidism and ear complaints to include those at high risk of developing MD. Extend awareness among hypothyroid patients about the importance of committing to hypothyroid medication and their risk of developing untreatable diseases like MD.

## 5 Limitations

Our study is burdened by several limitations. First, hypothyroidism has numerous etiologies that can be divided into several subgroups and included in the analysis. Second, other thyroid diseases, including hyperthyroidism, goiter, and thyroiditis should be included in future studies, they may have an impact on the occurrence of MD, due to the metabolic pathological changes and autoimmune nature these diseases have. Third, assessing patients' satisfaction with treatment, quality of life, and the effects of medication compliance and non-compliance warrants further prospective studies to be planned.

## 6 Conclusions

Many patients with hypothyroidism are diagnosed with MD. Clinicians should consider clinically screening for MD among hypothyroid patients presenting to clinics.

Thyroxine therapy could benefit aural symptoms and may prevent from MD. Further studies are needed to evaluate the efficacy of MD screening programs and thyroxine therapy in hypothyroid patients with MD symptoms.

## Availability of data and materials

All data related to this paper's conclusion are available and stored by the authors. All data are available from the corresponding author on reasonable request.

## Ethics approval and consent to participate

The work in this study complies with the principles laid down in the Declaration of Helsinki (Recommendations guiding physicians in biomedical research involving human subjects. Adopted by the 18th World Medical Assembly, Helsinki, Finland, June 1964, amended by the 29th World Medical Assembly, Tokyo, Japan, October 1975, the 35th World Medical Assembly, Venice, Italy, October 1983, and the 41st World Medical Assembly, Hong Kong, September 1989). This study was approved by the Institutional Review Board (IRB) at SPU. No reference number was given. Written consent was obtained from all participants. Participation in the study was voluntary and participants were assured that there would be no victimization of anyone who did not want to participate or who decided to withdraw after giving consent.

## Funding

This research received no specific grant from the Syrian Private University or any other funding agency in the public, commercial or non-profit sectors.

## Author contribution

Anan Bakdounes and Nawal Akashe were responsible for study design, literature search, and write up; Duaa Bakdounes participated in data collection; Mhd Obai Alchallah did the statistical analysis of data and contributed to the written paper; Homam Alolabi participated in the statistical analysis of data; Fatema Mohsen participated in the analysis and interpretation of data and wrote the final draft; Louei Darjazini Nahas participated in the study design and reviewed the final draft. All authors read and approved the final draft.

## Registration of research studies

1. Name of the registry: Prevalence of Ménière's Disease in Syrian Patients with Hypothyroidism
2. Unique Identifying number or registration ID: 10.5281/zenodo.6616112
3. Hyperlink to your specific registration (must be publicly accessible and will be checked): https://zenodo.org/record/6616112#.Yp9D-KjMI2w

## Guarantor

The Guarantors are Anan Bakdounes, Nawal Akashe, Mhd Obai Alchallah MD, Homam Alolabi MD, Duaa Bakdounes, Fatema Mohsen MD, and Louei Darjazini Nahas MD.

## Consent

Written consent was obtained from all participants. Participation in the study was voluntary and participants were assured that there would be no victimization of anyone who did not want to participate or who decided to withdraw after giving consent.

## Provenance and peer review

Not commissioned, externally peer reviewed.

## Financial disclosures/conflicts of interest

This study was received no funds. There are no conflicts of interest, financial, or otherwise.

## Declaration of competing interest

None.
